# Benthic habitat mapping of Plazh Gradina – Zlatna ribka (Black Sea) and Karpathos and Saria Islands (Mediterranean Sea)

**DOI:** 10.3897/BDJ.9.e71972

**Published:** 2021-08-23

**Authors:** Stamatina Nikolopoulou, Dimitar Berov, Stefania Klayn, Lyubomir I. Dimitrov, Kiril Velkovsky, Eva Chatzinikolaou, Giorgos Chatzigeorgiou, Ventzislav Karamfilov, Christina Pavloudi

**Affiliations:** 1 Hellenic Centre for Marine Research (HCMR), Institute of Marine Biology, Biotechnology and Aquaculture (IMBBC), 71500, Heraklion, Crete, Greece Hellenic Centre for Marine Research (HCMR), Institute of Marine Biology, Biotechnology and Aquaculture (IMBBC), 71500 Heraklion, Crete Greece; 2 Laboratory of Marine Ecology, Institute of Biodiversity and Ecosystem Research, Bulgarian Academy of Sciences, 2 Major Yurii Gagarin Street, 1113, Sofia, Bulgaria Laboratory of Marine Ecology, Institute of Biodiversity and Ecosystem Research, Bulgarian Academy of Sciences, 2 Major Yurii Gagarin Street, 1113 Sofia Bulgaria; 3 Institute of Oceanology, Bulgarian Academy of Sciences, 40 First May St, 9000, Varna, Bulgaria Institute of Oceanology, Bulgarian Academy of Sciences, 40 First May St, 9000 Varna Bulgaria; 4 Centre for underwater archaeology, Khan Krum sq. 1, 8130, Sozopol, Bulgaria Centre for underwater archaeology, Khan Krum sq. 1, 8130 Sozopol Bulgaria

**Keywords:** habitat mapping, marine benthic ecosystems, Karpathos, Saria, Sozopol, *
Posidonia oceanica
*, *
Cymodocea nodosa
*, *
Zostera noltei
*, *
Zostera marina
*, EUNIS, Mediterranean Sea, Black Sea

## Abstract

**Background:**

Habitat mapping is nеcessary for the efficient conservation and protection of marine ecosystems. In addition, it is a requirement for EU Member States as stated in the European Union (EU) Habitats Directive (92/43/EEC), as well as necessary for the achievement and maintenance of 'good environmental status (GES)' of benthic marine habitats in the framework of the EU Marine Strategy Framework Directive (2008/56/EC).

**New information:**

This study provides baseline information on the marine benthic habitats of Sozopol Bay (Black Sea) and Karpathos and Saria Islands (Mediterranean Sea). These two Natura 2000 sites were selected as study sites of the RECONNECT project, which aimed at creating a transnational cooperative network to confront the environmental threats of ecosystems with a high natural and cultural interest, by the establishment of common practices and a joint regional strategy. The specific objective was to map the marine habitats using a defined *a priori* classification (EUNIS), with the ultimate purpose of supporting government marine spatial planning, management and decision-making processes through the development of a Decision Support System.

## Introduction

Although the term habitat can be defined in a variety of ways (e.g. Bunce et al. 2013), it usually refers to a combination of living forms and abiotic factors (e.g. soil type and climate) occurring together (Ichter et al. 2014). According to the definition from the European Nature Information System (EUNIS) of the European Environment Agency (EEA), a habitat is "a place where plants or animals normally live, characterised primarily by its physical features (topography, plant or animal physiognomy, soil characteristics, climate, water quality, etc.), and secondarily by the species of plants and animals that live there" (Davies et al. 2004). Similarly, marine habitat mapping as defined by the Mapping European Seabed Habitats (MESH) project refers to "plotting the distribution and extent of habitats to create a map with complete coverage of the seabed showing distinct boundaries separating adjacent habitats" (Davies and Young 2008).

As required by the European Union (EU) Habitats Directive (92/43/EEC), EU Member States need to identify and designate sites for habitats (as listed in Annex I of the Directive) to be included in the Natura 2000 network. The same Directive requires EU Member States to report on the conservation status of these habitats at six-yearly intervals, which calls for solid knowledge of their geographical distribution. Therefore, it is clear that habitat mapping is a key feature of ecosystem management and conservation and it is a prerequisite for marine habitat restoration and adherence to the relative legislative framework (Gerovasileiou et al. 2019).

Regarding marine biomes, however, the focus over the last few years has been on the establishment of Marine Protected Areas (MPAs), which are the main tool for protection and conservation of the marine realm (Xuereb et al. 2019). Several concepts have been explored for the effective creation of MPAs, with the concept of Cells of Ecosystem Functioning (CEFs) being the most comprehensive one: the three-dimensional spaces where connections of intra- (e.g. life cycles), inter- (e.g. food webs) and extra-specific fluxes (e.g. biogeochemical cycles) are stronger than in adjacent areas (Boero et al. 2019). However, most of the MPAs in Europe, especially in the Mediterranean Sea, have been established for the protection of key species and do not take all the aforementioned connections thoroughly into account. In addition, they are of small size and are considered to be practically unsuccessful due to lack of efficient financial and political support (Barberá et al. 2012).

## General description

### Purpose

A successful regional habitat mapping programme needs to include the following elements (Ehrhold 2007):

- Clear statement of purpose for the mapping project (e.g. well defined goals and objectives).
- Selections of scales for map extents and data resolution appropriate to the stated purpose.
- A universally accepted and broadly applicable hierarchical habitat classification system, based on spatially nested physical and biophysical characteristics that control where species live.
- A means for acquiring data at appropriate resolutions and spatial scales for each of the relevant habitat characteristics.
- A means for combining, analysing and displaying these various geospatial datasets collected in diverse formats and at different scales and resolutions, such that the habitat classification system may be applied.

In accordance with these general guidelines, we identified the necessary steps for the selection of the mapping approach and methods that were applicable to our marine habitat mapping activities:

- Identification of the spatial extent of the mapping exercise.
- Determination of the objects to be mapped: substrate types and biological communities, as well as an appropriate system for the classification of these target types.
- Determination of the spatial resolution of the substrate mapping and biological communities mapping.
- Selection of appropriate methods for geophysical and biological sampling to fulfil these mapping goals with the available resources and time.
- Selection of appropriate data analysis tools and methods for habitat suitability modelling.

The aim of this study was to provide habitat maps for two Natura 2000 sites, one in Bulgaria and one in Greece, that were used for the creation of a decision-support system for the management of the sites as part of the “Interreg Balkan-Mediterranean 2014-2020” project RECONNECT. The most current EU-wide habitat classification systems for habitat classification with a thorough coverage of the unique benthic habitats of the two sites are the Habitats Directive classification scheme, the 2019 EUNIS classification system, the Marine Strategy Framework Directive (MSFD) Broad Habitat Types and the updated Barcelona Convention (BC) classification system (Montefalcone et al. 2021). The EU Habitat Directive habitat types that are recognised in the EU classification schemes have direct application in Natura 2000 area management and were the focus of our mapping activities.

## Sampling methods

### Study extent

**Figure 1.**
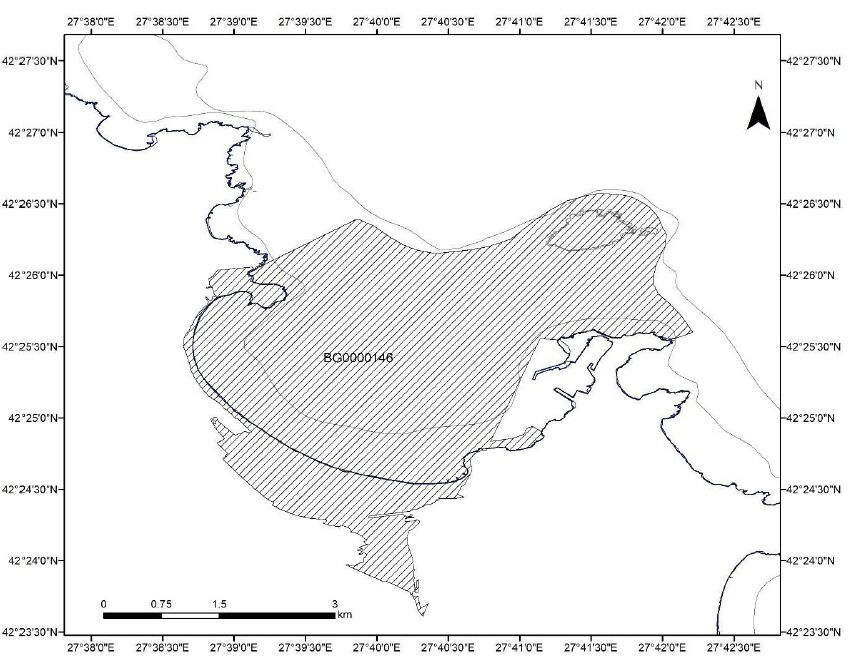
Spatial extent of the Plazh Gradina – Zlatna ribka Natura 2000 Protected Area (BG0000146).

The target of our mapping activities in Bulgaria was the maritime part of the Natura 2000 site BG0000146 Plazh Gradina – Zlatna ribka (Fig. 1). It has an area of 1033.5 ha, which includes 933.65 ha of the habitat type 1110 (Sandbanks slightly covered by seawater all the time), 95.92 ha of habitat 1170 (Reefs) and 4.5 ha of the habitat type 1140 (Mudflats and sandflats not covered by seawater). The main target for the mapping activities in the study area were the benthic biological communities.

**Figure 2.**
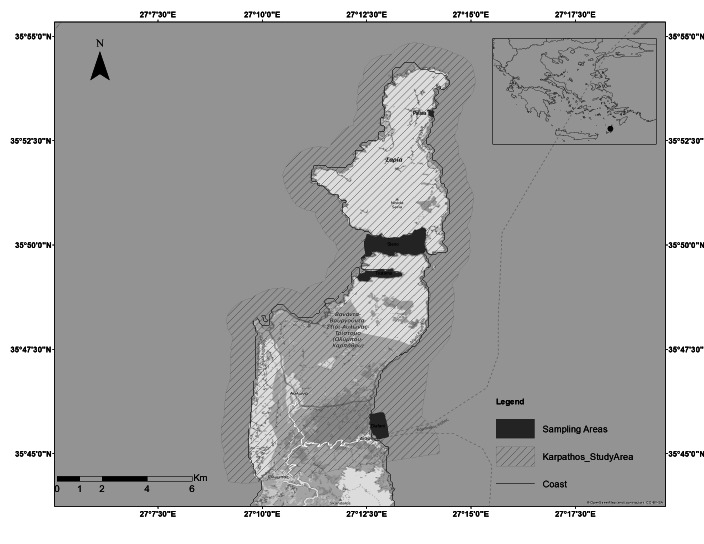
Spatial extent of the Natura 2000 Protected Area of northern Karpathos and Saria (GR4210003).

The targeted area in Greece was the Protected Area of northern Karpathos and Saria (GR4210003) (Fig. 2). Karpathos and the smaller Island Saria are located in the south-eastern Aegean Sea, bordering the Sea of Crete in the east and Rhodes Island in the southwest. A narrow strip of sea, no deeper than 80 metres, separates the two Islands. The Protected Area is considered to be one of the most important in Greece due to the existence of many rare and endemic species of flora and fauna. Its marine part has an area of 5181.74 ha, i.e. about 45% of its total area, which includes the habitat types 1110 (Sandbanks slightly covered by seawater all the time), 1120 (*Posidonia* beds (Posidonion oceanicae)), 1170 (Reefs), A5.531 (*Cymodocea* beds (*Cymodocea nodosa*)) and 8330 (Submerged or partially submerged sea caves). Karpathos hosts one of the largest populations of the Mediterranean monk seal (*Monachusmonachus*) (MOm 2009) and has been declared one of the ‘Important Bird Areas of Europe’ (Pipinos and Fokiali 2009).

### Sampling description

For the Natura 2000 site BG0000146:

The geophysical mapping of substrates and bathymetry were carried out by the Centre for Underwater Archaeology-Sozopol (K. Velkovsky, K. Dimitrov). The survey was set up in a 5 x 5 m grid with a resolution of < 1 m for the depth within each frame. The actual resolution of depth depended on the limitations of the side scan and multibeam profilers. The bathymetric survey was carried out with a multibeam sonar Teledyne Odom MB1 (512 beams), while bottom substrate texture types and relief were surveyed with a StarFish 450H side scan sonar. Both were mounted on a research vessel (MK ‘Hristina’, CUA-Sozopol) with a high precision GPS system (RTK DGPS Trimble). The survey vessel performed transects in the study area, parallel to the shoreline and with at least 20% overlap of the scanned area of the bottom between two adjacent transect lines. The survey covered the sea bottom down to 3 m depth, avoiding any shallow areas and obstacles in the zone. The depths and topography of the areas shallower than 3 m were later interpolated, based on available data from previous surveys. All data were continuously recorded on-board the vessel using computers with the appropriate software systems.

The sonograms of the scanned area were later used for the creation of a substrate type map of the zone. This mapping was based on previous geological substrate sampling campaigns in the area, carried out by the Institute of Oceanology-BAS (IO-BAS, L. Dimitrov). The substrate texture types were classified and matched to the Folk-16 substrate classification scheme, based on data from transects with over 160 point samples. This included several categories оf rocky substrates, soft bottom substrates (sand and mud), soft bottom substrates with seagrasses and artificial substrates (concrete, pipelines etc.).

The results from the geophysical mapping were ground-truthed with benthic macrofaunal samples, video and still image drop cameras in a sampling resolution sufficiently dense to cover the different sediment types and depth ranges in the study area, following the methodology described in Karamfilov et al. 2017). A 250 x 250 m sample grid was established within the study area; at least one sample/photo/video was taken within each of these sampling squares, ensuring a minimum of 1:25,000 resolution of the *in situ* biological data. In areas with higher heterogeneity of habitats, where more than one habitat type was expected to be present within each square (e.g. the rocky coastal zone between 0-1 and 10 m), more than one sample and video/still image observations were taken per square.

Data analysis was performed with the following software products: HyPack for bathymetry data, Deep View for the side scan sonar and mosaic data and Global Mapper for the creation of substrate and overview maps. The resulting data were saved as DEM, XYZ, GeoTIFF (3D format) for the terrain models and GeoTIFF and georeferenced JPGs for the side scan data and the collected substrate type data.

Georeferenced photos of the benthic communities were analysed by experienced benthic ecologists, identifying the substrate types, dominant macroalgal and zoobenthic species visible in the photo and the dominant habitat types, in accordance with the EUNIS 2019 (level 3 and 4) classification system, as well as the Bulgarian national MSFD habitat types and subtypes. Results were imported in ArcMap 10.4 and the spatial distribution of habitat types was further explored there, by determining the depth limit of distribution of habitats and dominant species on the detailed substrate and bathymetry map of the study site.

For the Natura 2000 site GR4210003:

Bottom substrates were surveyed with a DeepVision (DE3468D) portable side scan sonar trawled on the “Saria” vessel, equipped with a high precision GPS system (GPSMAP78, Garmin). The survey vessel performed a number of transects in the study sub-areas, in most cases parallel to the shoreline and with 5-15% overlap of the scanned area of the bottom between two adjacent transect lines (depending on the shoreline and the weather conditions). The scanning frequency was 680 kHz. All data were continuously recorded onboard the vessel using a computer with the appropriate software systems installed. Additionally, the BlueRobotics, BLUE ROV2 (ver. heavy duty) underwater drone was used for ground-truthing of the different habitat types. The sonograms of the scanned areas were used for the creation of substrate type maps of the area. Data analysis was performed with the software products DeepView Pro for the side scan sonar data and QGIS3.12 for the creation of substrate and overview maps. Data were exported in KML format by DeepView, transformed into shape files, projected in UTM35 and processed in QGIS on a map scale 1:20,000. Polygons of the same habitat were merged so each feature within the shapefile was assigned to one habitat (1110 soft bottom, 1120 *Posidonia*, 1170 Hard substrate and A5.531 *Cymodocea* beds).The process of habitat delimiting was based on the analysis of the geophysical and sedimentology data, where borders between sediment subtypes and substrate types were drawn (manually), based on *in situ* sampling data confirming difference in substrates in areas with similar side scan acoustic images texture and characteristics.

### Quality control

To ensure that the data would display properly, the shapefiles were cleaned by removing any geometry or topology errors. The shape length and area were calculated in metres and square metres, respectively and all habitats were visualised via Geoserver on MedOBIS viewer (available at https://portal.lifewatchgreece.eu/, accessible after registration) in WGS84 coordinate system (EPSG:4326). Downloading in .csv and .KML format is available through the 'Save WMS Layer" tool. An OGC standard for geospatial styling (Styled Layer Descriptor (SLD)) was used through Geoserver to create styles for each layer. Open Street Map was used as a base-map, as it is an up-to-date open source map (Figs 3, 4).

The length and total area of each habitat type in each of the Natura 2000 sites are given in Table 1 and Fig. 5.

Data quality assurance procedures included in the EMODnet Seabed Habitats data submission process were followed during the preparation of the final datasets. Тhe confidence assessment procedure included an evaluation of the quality, spatial coverage and precision of geographical positioning of the used remote sensing techniques and ground-truthing methods, as well as assessments of the quality, accuracy and representative nature of the produced maps of biological communities. The evaluation results are included in the metadata files of the produced shape files.

## Geographic coverage

### Description

For the Natura 2000 site BG0000146:

The official name is Plazh Gradina - Zlatna ribka; located in the Black Sea, with an area of 12 km^2^; it is a Protected Area under Directive 92/43/EEC for the conservation of natural habitats and of wild fauna and flora. The site includes the Island of St. Ivan and the small Island of St. Peter next to it; the whole bay is called Sozopol. The latter is a beautiful coastal town founded in 611 BC. Sampling area: 14,091,859.35 m^2^ (14 km^2^, 1,409 ha), of which 3.2% is *Zostera* spp. (*Zosteranoltei* and *Zosteramarina*) meadows. Depth range: 0-30 m. The sampling area stretches between 42.408660 and 42.449459 Latitude; 27.645078 and 27.706018 Longitude.

An official designation order with detailed management plans and restrictions for the area was published by the Bulgarian Ministry of Environment and Water in May 2021. It includes management measures and regulations that were suggested by IBER-BAS as a result of the surveys completed in this study (Bulgarian State Gazette, Issue 45/28/05/2021).

For the Natura 2000 site GR4210003:

The selected study sites covered Tristomo and Diafani Bay in Karpathos, Palatia in Saria Island and Steno (Diaplous), the narrow area between Saria and Karpathos. The sampling area stretches between 35.755691 and 35.887419 Latitude; 27.204275 and 27.235387 Longitude.

Diafani is a traditional fishing village, the second port of the Island and is located in the northeast of the Island. Sampling area: 647,334.95 m^2^ (65 ha), of which 29% is *Posidoniaoceanica* meadows. Depth range: 0-20 m.

Tristomo is an enclosed bay, a natural harbour, separated from the open sea by three openings, located in the northern part of Karpathos. Sampling area: 479,947.20 m^2^ (48 ha), of which 5.4% is *Posidoniaoceanica* meadows. Depth range: 0-25 m.

Steno (Diaplous) is the channel between the Islands of Karpathos and Saria. Sampling area: 1,867,246.19 m^2^ (187 ha), of which 6% is *Posidoniaoceanica* meadows. Depth range: 0-82 m.

Palatia is a bay in Saria Island. Sampling area: 53,539.28 m^2^ (5 ha), of which 17% is *Posidoniaoceanica* meadows. Depth range: 0-41 m.

### Coordinates

35.2991739963 and 42.5214755477 Latitude; 27.0079103795 and 27.9142824498 Longitude.

## Temporal coverage

**Data range:** 2019-11-09 – 2019-11-17.

### Notes

Primary data from the Islands of Karpathos and Saria were collected in one visit in November 2019 (09/11/2019 - 17/11/2019). Data for the 'Plazh Gradina - Zlatna ribka' site were collected in a series of field studies carried out between June 2018 and October 2019.

## Usage licence

### Usage licence

Other

### IP rights notes

Attribution-NonCommercial-ShareAlike 4.0 International (CC BY-NC-SA 4.0)

## Data resources

### Data package title

Habitats of Natura 2000 Protected Areas BB0000146 and GR4210003

### Number of data sets

2

### Data set 1.

#### Data set name

Soft bottom and hard bottom habitats within the BB0000146 'Gradina-Zlatna ribka' SCI area

#### Number of columns

7

#### Download URL

https://www.seanoe.org/data/00665/77683/

#### 

**Data set 1.** 

Column label	Column description
POLYGON	Unique identifier for each polygon
GUI	Globally unique identifier of the habitat map. It consists of a 2-letter country code + 6 digits
ORIG_HAB	The information of the habitat type present in the polygon, based on Eunis Habitat classification system
ORIG_CLASS	Brief description of the habitat classification system
COMP	A description of the habitats within polygon groups
COMP_TYPE	The type of composition for the habitats within the polygon group. "single habitat" - the polygon is not within a group
Area	The shape area in square metres or hectares

### Data set 2.

#### Data set name

Habitat maps of the Protected Area of northern Karpathos and Saria (GR4210003) (December 2020)

#### Data format

Shapefile

#### Number of columns

7

#### Character set

UTF-8

#### Download URL

https://www.seanoe.org/data/00666/77813/

#### Description

**Data set 2.** 

Column label	Column description
POLYGON	Unique identifier for each polygon
GUI	Globally unique identifier of the habitat map. It consists of a 2-letter country code + 6 digits
AnnexI	Official habitat code of Annex I
ORIG_HAB	The information of the habitat type present in the polygon, based on Eunis Habitat classification system
CONFIDENCE	Confidence in presence and extent of habitat
ORIG_CLASS	Brief description of the habitat classification system
COMP_TYPE	The type of composition for the habitats within the polygon group. "single habitat" - the polygon is not within a group

## Additional information

The dataset for the Natura 2000 site BG0000146 is also available at:

http://gis.ices.dk/geonetwork/srv/eng/catalog.search#/metadata/0bf67a42-b478-4ac5-b0df-3e5e9aa638df

The dataset for the Natura 2000 site GR4210003 is also available at:

http://gis.ices.dk/geonetwork/srv/eng/catalog.search#/metadata/5877d808-3fd7-11eb-b378-0242ac130002

## Figures and Tables

**Figure 3a.**
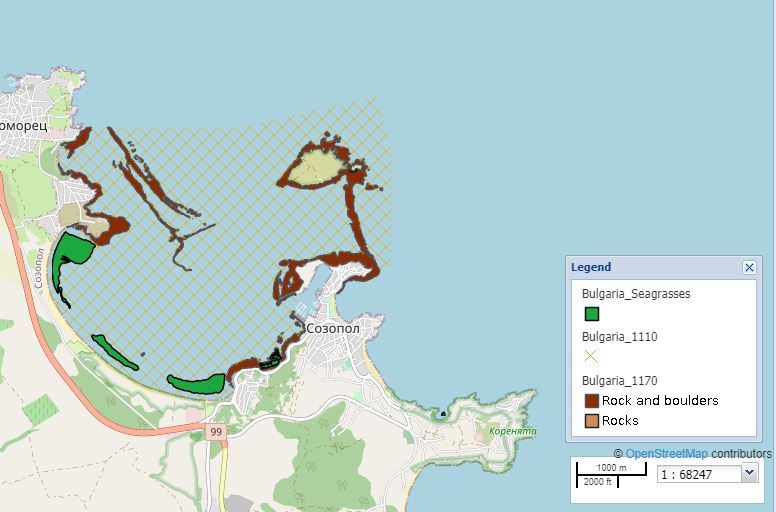
Habitat distribution map of the study area.

**Figure 3b.**
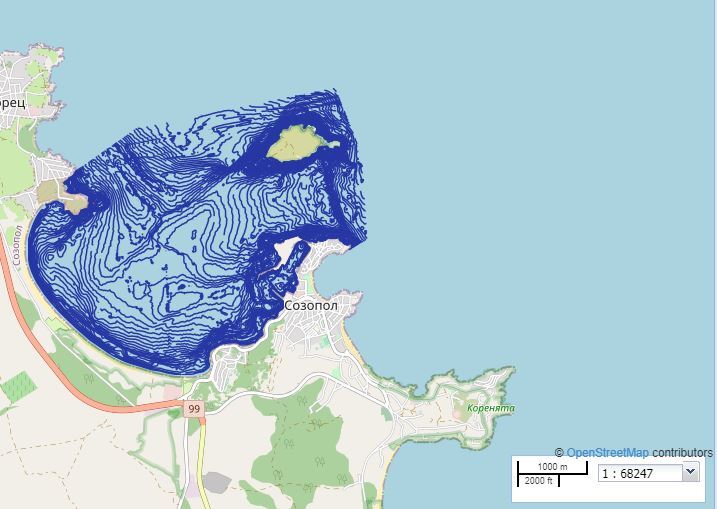
Bathymetric map of the study area.

**Figure 4a.**
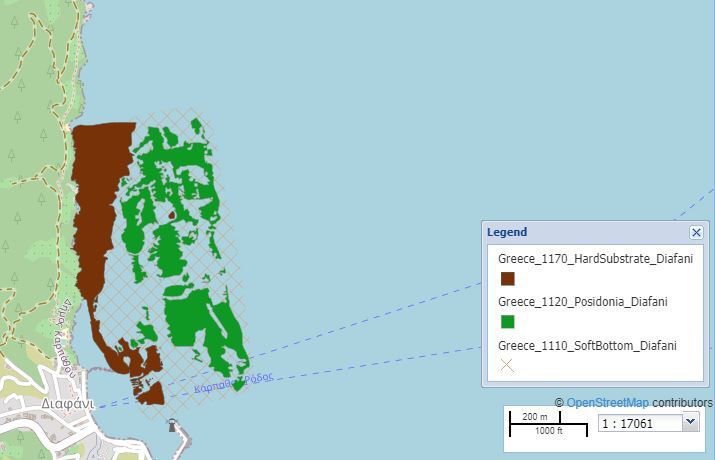
Map of the Diafani area.

**Figure 4b.**
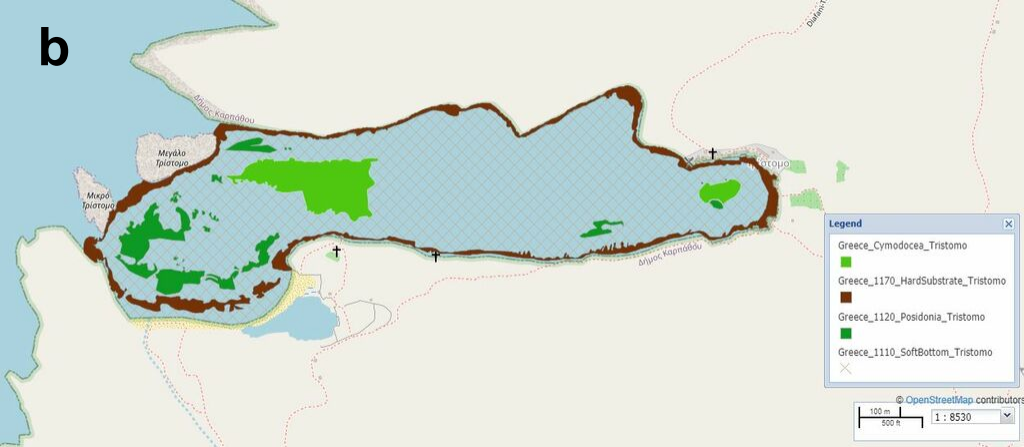
Map of the Tristomo area.

**Figure 4c.**
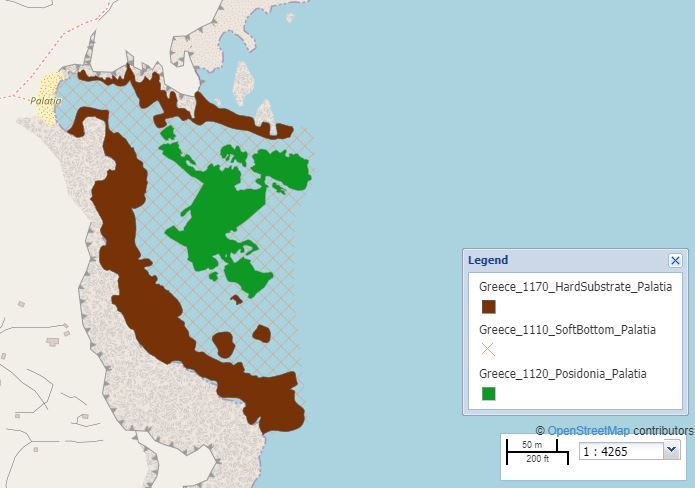
Map of the Palatia area.

**Figure 4d.**
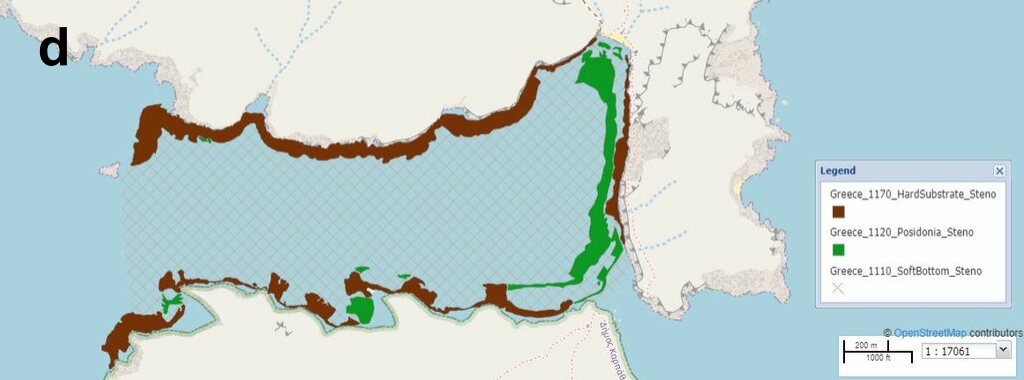
Map of the Steno (Diaplous) area.

**Figure 5a.**
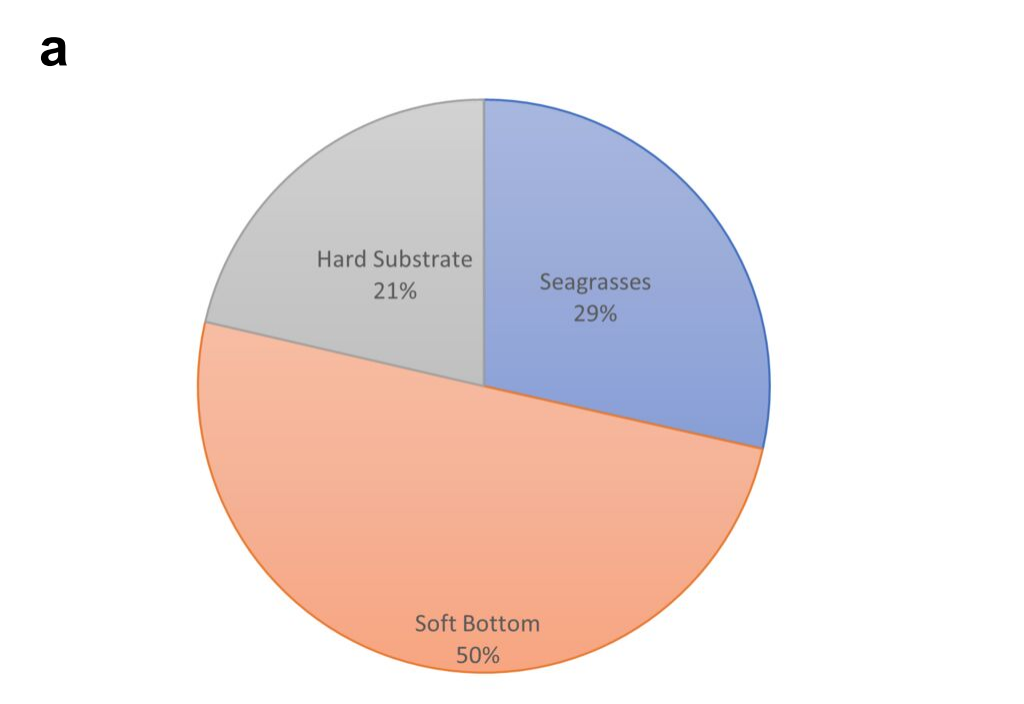
Percentages of the shape area of the main habitat types at Diafani.

**Figure 5b.**
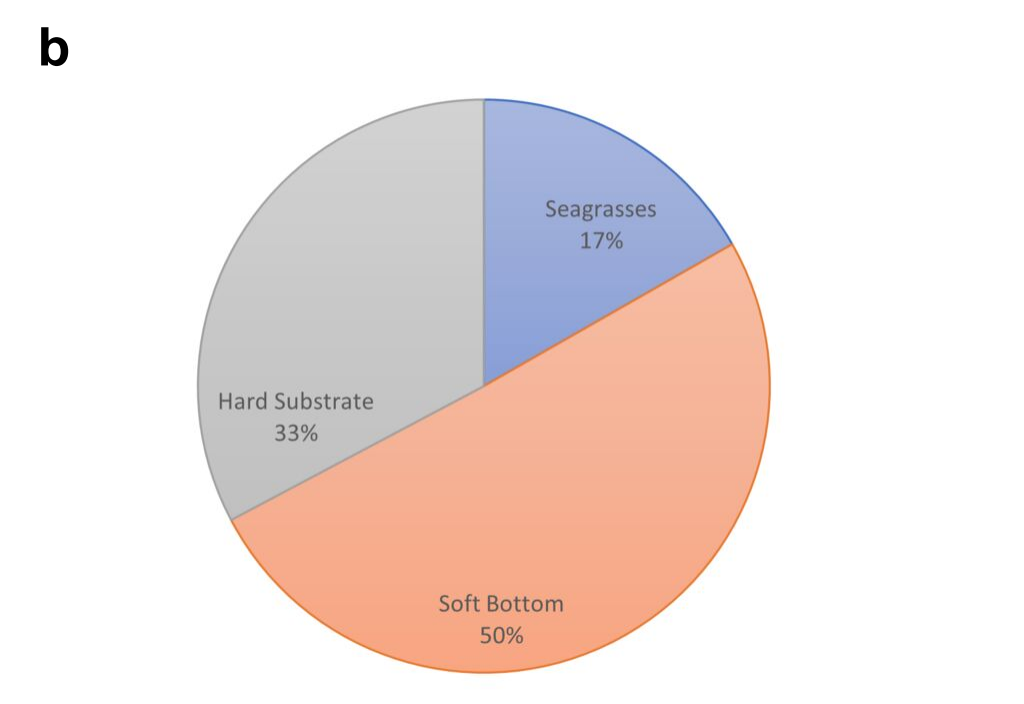
Percentages of the shape area of the main habitat types at Palatia.

**Figure 5c.**
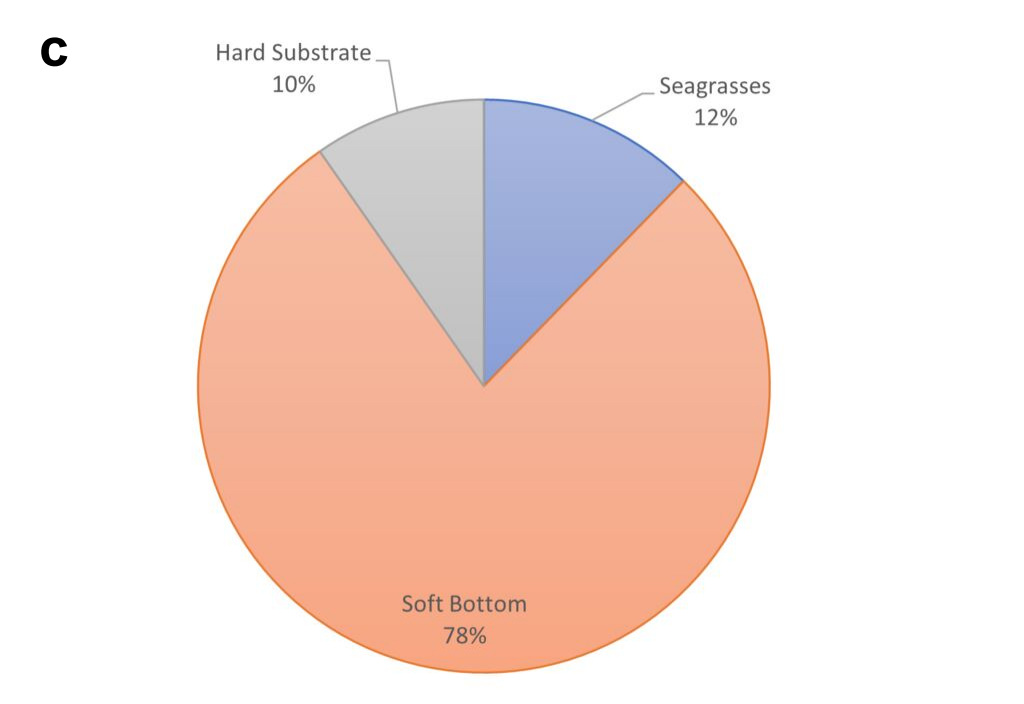
Percentages of the shape area of the main habitat types at Tristomo.

**Figure 5d.**
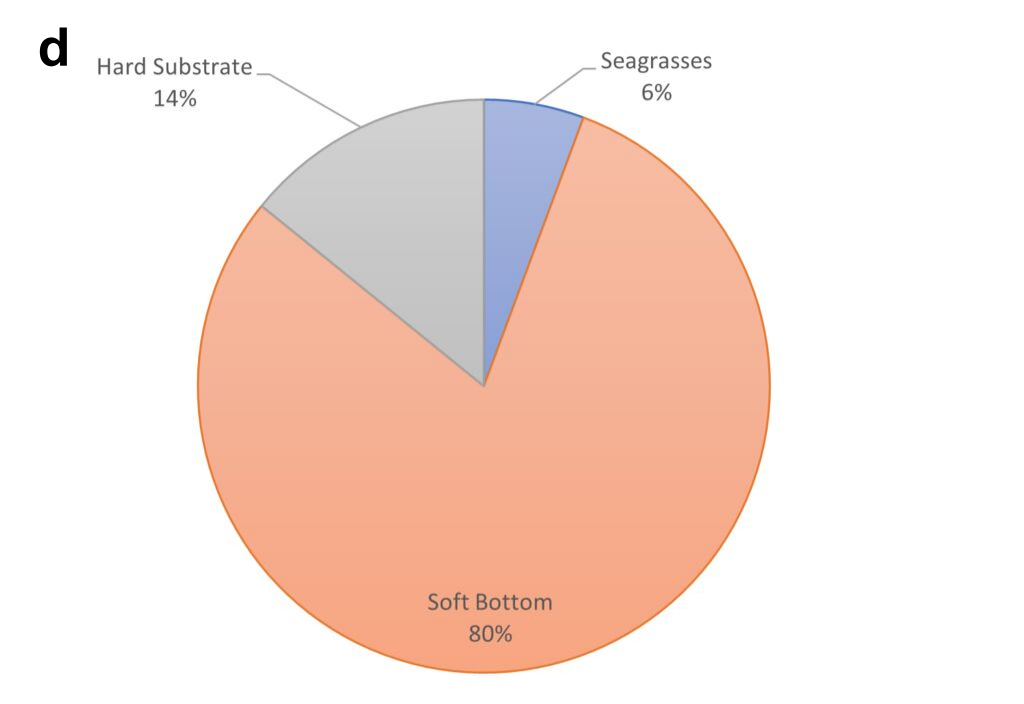
Percentages of the shape area of the main habitat types at Steno (Diaplous).

**Figure 5e.**
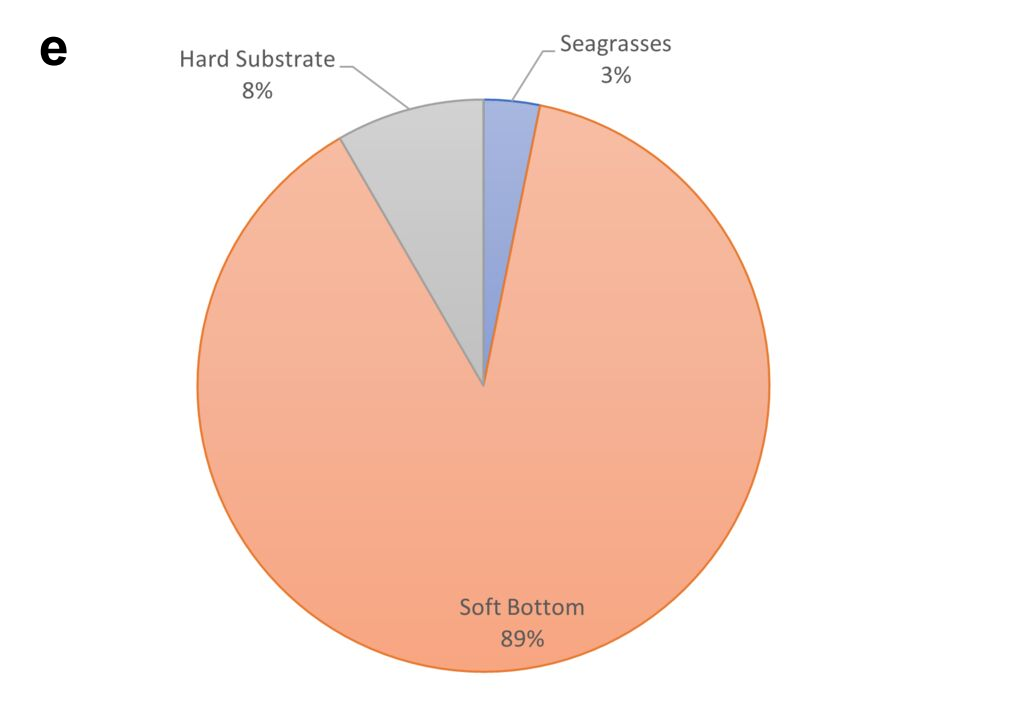
Percentages of the shape area of the main habitat types at Sozopol Bay.

**Table 1.** The shape area of each habitat type in each Natura 2000 site, as downloaded from the MedOBIS viewer.

		Seagrasses (*Posidoniaoceanica*, *Cymodoceanodosa*, *Zostera* spp.)	Soft Bottom	Hard Substrate
		Shape area (m^2^)	Shape area (m^2^)	Shape area (m^2^)
GR4210003	Palatia	8,970.28	27,041.06	17,527.23
	Diafani	184,620.62	324,222.95	138,491.40
	Steno (Diaplous)	105,808.65	1,496,775.74	264,661.80
	Tristomo	59,073.53	374,203.55	46,670.13
BG0000146		448,757.20	12,465,103.14	1,179,832.27
